# Antibacterial activities and structure–activity relationships of a panel of 48 compounds from Kenyan plants against multidrug resistant phenotypes

**DOI:** 10.1186/s40064-016-2599-1

**Published:** 2016-06-27

**Authors:** Leonidah K. Omosa, Jacob O. Midiwo, Armelle T. Mbaveng, Simplice B. Tankeo, Jackson A. Seukep, Igor K. Voukeng, Joachim K. Dzotam, John Isemeki, Solomon Derese, Ruth A. Omolle, Thomas Efferth, Victor Kuete

**Affiliations:** Department of Chemistry, School of Physical Sciences, University of Nairobi, P. O. Box 30197-00100, Nairobi, Kenya; Department of Biochemistry, Faculty of Science, University of Dschang, Dschang, Cameroon; Department of Pharmaceutical Biology, Institute of Pharmacy and Biochemistry, Johannes Gutenberg University, Staudinger Weg 5, 55128 Mainz, Germany

**Keywords:** Anthraquinones, Benzoquinones, Chalcones, Antibacterial activities, Multidrug resistance, Efflux pump inhibitor

## Abstract

In the current study forty eight compounds belonging to anthraquinones, naphthoquinones, benzoquinones, flavonoids (chalcones and polymethoxylated flavones) and diterpenoids (clerodanes and kauranes) were explored for their antimicrobial potential against a panel of sensitive and multi-drug resistant Gram-negative and Gram-positive bacteria. The minimal inhibitory concentration (MIC) determinations on the tested bacteria were conducted using modified rapid INT colorimetric assay. To evaluate the role of efflux pumps in the susceptibility of Gram-negative bacteria to the most active compounds, they were tested in the presence of phenylalanine arginine β-naphthylamide (PAβN) (at 30 µg/mL) against selected multidrug resistance (MDR) bacteria. The anthraquinone, emodin, naphthaquinone, plumbagin and the benzoquinone, rapanone were active against methicillin resistant *Staphylococcus aureus* (MRSA) strains of bacteria with MIC values ranging from 2 to 128 μg/mL. The structure activity relationships of benzoquinones against the MDR Gram-negative phenotype showed antibacterial activities increasing with increase in side chain length. In the chalcone series the presence of a hydroxyl group at C3′ together with a methoxy group and a second hydroxyl group in *meta* orientation in ring B of the chalcone skeleton appeared to be necessary for minimal activities against MRSA. In most cases, the optimal potential of the active compounds were not attained as they were extruded by bacterial efflux pumps. However, the presence of the PAβN significantly increased the antibacterial activities of emodin against Gram-negative MDR *E. coli* AG102, 100ATet; *K. pneumoniae* KP55 and KP63 by >4–64 g/mL. The antibacterial activities were substantially enhanced and were higher than those of the standard drug, chloramphenicol. These data clearly demonstrate that the active compounds, having the necessary pharmacophores for antibacterial activities, including some quinones and chalcones are substrates of bacterial efflux pumps and therefore should be combined to efflux pump inhibitors in the fight against MDR bacterial infections.

## Background

Multidrug resistance (MDR) in bacteria is usually mediated by the expression of efflux pumps or porins involved in transport, by the expression of mutated genes coding for specific drug targets or specific enzymatic barriers. As a matter of fact, MDR remains a major obstacle hindering successful antibacerial chemotherapy (Alekshun and Levy 2007; Davin-Regli et al. 2008; Nikaido 2009).

One way of tackling the emergence of MDR is to diversify the chemical structures of anti-microbial drugs to which resistance has developed in order to extend their lifespan (De Clercq 2001; Poole 2001; Jeu et al. 2003). Alternatively, the compounds exhibiting modest to significant antibacterial activities against MDR phenotypes, could be used in combination with the efflux inhibitors, in order to improve on the accumulation of the drug in the cells for optimal activities (Kuete et al. 2011). It is not surprising that in response to anti-microbial resistance, major pharmaceutical companies have concentrated their efforts on improving chemotherapeutic agents in established drug classes (Taylor et al. 2002). However, with the portfolio of antimicrobials currently available, most of the lead structures of current drugs have already been explored. Today, large-scale empirical screening of synthetic, semi-synthetic and natural chemical entities in chemical libraries for anti-microbial activities is investigated (Kimberlin and Whitley 1996). The compounds of interest for the development of novel drugs include those that target different proteins or biochemical processes compared to those in current use and those that inhibit bacterial efflux pumps (Kuete et al. 2011). Natural products have been a particularly rich source of anti-infective agents, yielding, for example, the penicillins in 1940, the tetracyclines in 1948 and the glycopeptides in 1955 (Silver and Bostian 1990). In the current study the antibacterial activities of compounds from several families (anthraquinones, naphthoquinones, benzoquinones, chalcones, flavones, flavanones, clerodane, and kaurane diterpenoids) were determined against different bacterial strains expressing MDR phenotypes. Furthermore, the chemical moieties relevant for pharmacophore binding were analyzed by their structure activity relationships (SAR) studies of related compounds.

## Results and discussion

### Studied compounds

**Fig. 1:**
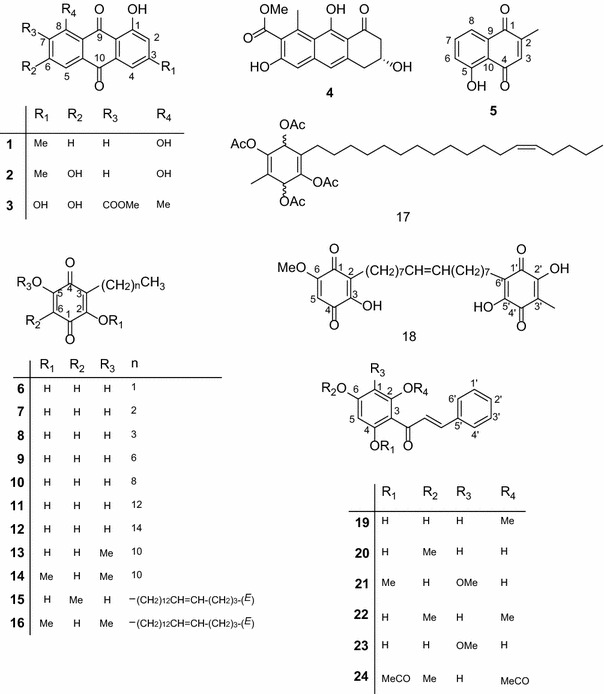
Chemical structures of the compounds tested; **1**, chrysophanol; **2**, emodin; **3**, 3,6,8-trihydroxy-1-methylanthraquinone-2-carboxylic acid; Me; **4**, aloesaponol I; **5**, plumbagin; **6**, benzoquinones; 2,5-dihydroxy-3-ethyl-2,5-cyclohexadiene-1,4-dione; **7**, 2,5-dihydroxy-3-propyl-2,5-cyclohexadiene-1,4-dione; **8**, 2,5-dihydroxy-3-butyl-2,5-cyclohexadiene-1,4-dione; **9**, 2,5-dihydroxy-3-heptyl-2,5-cyclohexadiene-1,4-dione; **10,** homoembelin; **11**, rapanone; **12**, 2,5-dihydroxy-3-pentadecyl-2,5-cyclohexadiene-1,4-dione; **13**, 5-*O*-methylembelin; **14**, 2,5-di-*O*-dimethylembelin; **15**, maesaquinone; **16**, 2,5 dimethoxy-6-(14-nonadecenyl)-1,4-benzoquinone; **17**, 1,2,4,5-tetraacetate-3-methyl-6-(14-nonadecenyl)-cyclohexadi-2,5-diene; **18**, ardisiaquinone; **19**, 3′,5′-dihydroxy-1′-methoxychalcone; **20**, 1′,5′-dihydroxy-3′-methoxychalcone; **21**, 1′,3′-dihydroxy-2′,5′-dimethoxychalcone; **22**, 5′-hydroxy-1′,3′-dimethoxychalcone; **23**, 1′,3′,5′-trihydroxy-2′-methoxychalcone; **24**, 1,5-diacetate-3′-methoxychalcone

**Fig. 2:**
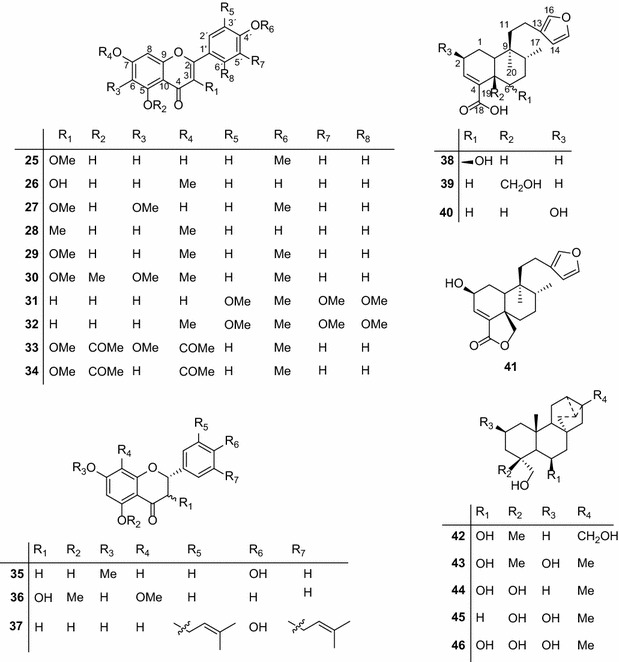
Chemical structures of the compounds tested; **25**, 5,7-dihydroxy-3,4-dimethoxyflavone; **26**, 3,5,4′-trihydroxy-7 methoxyflavone; **27**, 5,7-dihydroxy-3,6,4′-trimethoxyflavone; **28**, 5,4′-dihydroxy-3,7-dimethoxyflavone; **29,** 5-hydroxy-3,7,4′-trimethoxyflavone; **30**, 3,5,6,7,4′-pentamethoxyflavone; **31**, 5-hydroxy-2′,3′,4′,5′-tetramethoxyflavone; **32**, 5-hydroxy-7,2′,3′,4′,5′-pentamethoxyflavone; **33**, 5,7-diacetate-3,6,4′-trimethoxyflavone; **34**, 5,7-diacetate-3,4′-trimethoxyflavone; **35**, 5,4′-dihydroxy-7-methoxyflavanone; **36**, 3,7-dihydroxy-5,8-dimethoxyflavanone; **37**, 5,7,4′-trihydroxy-3′-prenylflavanone; **38**, dodonic acid; **39**, hautriwaic acid; **40**, 2β-hydroxyhardwickiic acid; **41**, hautriwaic acid lactone; **42**, 6,17,19-trachylobanetriol; (ent-6α)-form; **43**, 2,6,19-trachylobanetriol; (ent-2α,6α)-form; **44**, 6,18,19-trachylobanetetrol; (ent-6α)-form; **45**, 2,18,19-trachylobanetriol; (ent-2α)-form; **46**, 2,6,18,19-trachylobanetetrol; (ent-2β,6α)-form; **47**, *ent*-kaur-16-en-2α,18,19-triol; **48**, *ent*-kaur-16-en-18,19-diol

The natural and modified compounds investigated in the current study were previously isolated from Kenyan plants (Figs. 1, 2). These included quinones; four anthraquinones namely; chrysophanol (**1**) (Fairbairn and El-Muhtadi 1972; Midiwo and Rukunga 1985; Zhang et al. 2012), emodin (**2**) (Munavu et al. 1984; Chang et al. 1996), 3,6,8-trihydroxy-1-methylanthraquinone-2-carboxylic acid; Me ester (**3**) (Mehandale et al. 1968; Dagne et al. 1996), aloesaponol I; (-)-form (**4**) (Yagi et al. 1978; Dagne et al. 1992; Midiwo et al. 1997), a naphthoquinones; 5-hydroxy-2-methyl-1,4-naphthalenedione, plumbagin (**5**) (Sidhu et al. 1968; Yuan and Chao 2007), thirteen related benzoquinones; 2,5-dihydroxy-3-ethyl-2,5-cyclohexadiene-1,4-dione (**6**) (Khurana et al. 1972) and synthetic derivatives, 2,5-dihydroxy-3-propyl-2,5-cyclohexadiene-1,4-dione (**7**), 2,5-dihydroxy-3-butyl-2,5-cyclohexadiene-1,4-dione (**8**), 2,5-dihydroxy-3-heptyl-2,5-cyclohexadiene-1,4-dione (**9**), 2,5-dihydroxy-3-nonyl-2,5-cyclohexadiene-1,4-dione, homoembelin (**10**) (Murthy et al. 1965), 2,5-dihydroxy-3-tridecyl-2,5-cyclohexadiene-1,4-dione, rapanone (**11**) (Wouters and Verhecken 1987), 2,5-dihydroxy-3-pentadecyl-2,5-cyclohexadiene-1,4-dione (**12**) (Ogawa and Natori 1965, 1968a), 2-hydroxy-5-methoxy-3-undecyl-1,4-benzoquinone, 5-*O*-methylembelin (**13**) (Merian and Schlittler 1948), 2,5-dimethoxy-3-undecyl-1,4-benzoquinone, 2,5-di-*O*-dimethylembelin (2,5-dimethoxy-3-undecyl-[1,4]-benzoquinone) (**14**) (Wu et al. 2009), 2,5-dihydroxy-3-methyl-6-(14-nonadecenyl)-1,4-benzoquinone, maesaquinone (**15**) (Ogawa and Natori 1965, 1968a; Ogawa and Natori 1968b; Manguro et al. 2003), 2,5 dimethoxy-6-(14-nonadecenyl)-1,4-benzoquinone (**16**), 1,2,4,5-tetraacetate-3-methyl-6-(14-nonadecenyl)-cyclohexadi-2,5-diene (**17**), ardisiaquinone (**18**) (Yoshihir et al. 1968; Ogawa and Natori 1968b), flavonoids including six chalcones; 3′,5′-dihydroxy-1′-methoxychalcone (**19**), 1′,5′-dihydroxy-3′-methoxychalcone (**20**), 1′,3′-dihydroxy-2′,5′-dimethoxychalcone (**21**), 5′-hydroxy-1′,3′-dimethoxychalcone (**22**), 1′,3′,5′-trihydroxy-2′-methoxychalcone (**23**), 1,5-diacetate-3′-methoxychalcone (**24**) (Midiwo et al. 1990; 1992), ten polymethoxylated flavones and their semi-synthetic derivatives; 5,7-dihydroxy-3,4-dimethoxyflavone (**25**), 3,5,4′-trihydroxy-7 methoxyflavone (**26**), 5,7-dihydroxy-3,6,4′-trimethoxyflavone (**27**), 5,4′-dihydroxy-3,7-dimethoxyflavone (**28**), 5-hydroxy-3,7,4′-trimethoxyflavone (**29**) (Omosa et al. 2010), 3,5,6,7,4′-pentamethoxyflavone (**30**), 5-hydroxy-2′,3′,4′,5′-tetramethoxyflavone (**31**), 5-hydroxy-7,2′,3′,4′,5′-pentamethoxyflavone (**32**) (Juma et al. 2001), 5,7-diacetate-3,6,4′-trimethoxyflavone (**33**) semi-synthetic derivative of 5,7-dihydroxy-3,6,4′-trimethoxyflavone (Omosa et al. 2010), 5,7-diacetate-3,4′-trimethoxyflavone (**34**) a semi-synthetic derivative of 5,7-dihydroxy-3,4′-trimethoxyflavone; three flavanones; 5,4′-dihydroxy-7-methoxyflavanone (**35**) (Kerubo et al. 2013), 3,7-dihydroxy-5,8-dimethoxyflavanone (**36**), 5,7,4′-trihydroxy-3′-prenylflavanone (**37**) (Nakahara et al. 2003); eleven diterpenoids, four clerodane type; dodonic acid (**38**), hautriwaic acid (**39**), 2β-hydroxyhardwickiic acid (**40**), hautriwaic acid lactone (**41**) (Omosa et al. 2010), five trachylobane type; 6,17,19-trachylobanetriol; (ent-6α)-form (**42**) (Juma et al. 2006), 2,6,19-trachylobanetriol; (ent-2α,6α)-form (**43**) (Midiwo et al. 1997), 6,18,19-trachylobanetetrol; (ent-6α)-form (**44**), 2,18,19-trachylobanetriol; (ent-2α)-form (**45**), 2,6,18,19-trachylobanetetrol; (ent-2β,6α)-form (**46**) (Juma et al. 2006) and two kaurane type; *ent*-kaur-16-en-2α,18,19-triol (**47**), *ent*-kaur-16-en-18,19-diol (**48**) (Midiwo et al. 1997) Figs. 1 and 2.

The compounds were tested for their ability to prevent the growth of MDR and reference strains of Gram-negative bacteria, alone and for some of the active compounds, in the presence of the efflux pump inhibitor (EPI), PAβN (Table 2).

The antibacterial activity of compounds has been defined as significant when the MIC is below 10 µg/mL, moderate when 10 < MIC < 100 µg/mL and low when MIC > 100 µg/mL (Kuete 2010; Kuete and Efferth 2010). Compound **1** was inactive against all drug sensitive and resistant bacteria. However, **2** with a similar skeletal structure as **1** except for the presence of an hydroxyl group at C6 was more active exhibiting antimicrobial activities against Gram-negative *E. coli* A102 and AG 100ATet; *K. pneumoniae* KP55, KP63, *E. aerogenes* EA289 with MIC values of 128, 16, 32, 128 and 128 µg/mL, respectively. This anthraquinone showed good activities against MRSA 4, 6 and 8 with MIC values of 4 (vs 8), 4 (vs 64), 4 (vs 32) μg/mL, respectively, more active than the standard drug, chloramphenicol. These results are comparable to those obtained by Hatano et al. (1999), where emodin exhibited noticeable antibacterial effects against four MRSA strains (OM481, OM505, OM584, OM623) and one MRSA strain (209P) with MIC values of about 64 μg/mL but less sensitive against the Gram-negative strains, *E. coli* K12 and *Pseudomonas aeruginosa* PA01 with MIC > 128 μg/mL. The presence of an hydroxyl group in place of a methyl group at C3 or a methyl in place of hydroxyl group at C8 and an additional methyl ester (COOMe) group at C7 in **3** substantially reduced antimicrobial activities especially against the MRSA phenotype, as this compound did not inhibit these bacteria. However, this compound exhibited minimal antimicrobial activities against the standard *E. coli* ATCC8739 strain with MIC values of 256 µg/mL, which was not inhibited by **2**. Compound **4,** which is a derivative of **3** with a slightly different skeletal structure in ring A had antimicrobial activities similar to those of **3**, probably due to the total number of hydroxyl groups (3), methyl (1) and acetate irrespective of the positions of these substituents in the anthroquinone skeleton. Furthermore, the substitution pattern of ring C was similar in the two compounds. This compound was also inactive against all bacteria strains tested except for the reference *E. coli* ATCC8939 strain with a MIC value of 256 µg/mL.

The naphthoquinone, plumbagin (**5**) was active against both Gram-positive and Gram-negative bacteria tested with interesting MIC values ranging from 2 to 64 μg/mL. This naphthoquinone exhibited exceptionally good antimicrobial activities against MRSA 3, 4, 6, 8 compared to chloramphenicol with MIC values of 64 (vs >256), 2 (vs 8), 2 (vs 64) and 2 (vs 32) µg/mL, respectively. The good antibacterial activity of this compound is consistent to data previously documented (Kuete et al. 2011). Several studies have also demonstrated the potencies of plumbagin against bacteria and fungi (Brice 1955; Durga et al. 1990; Gujar 1990) as well as cancer (Melo et al. 1974). In a separate study the in vitro antimicrobial activities of plumbagin against selected microorganisms were reported to be significantly higher than the standard drug, streptomycin (Jeyachandran et al. 2009). The antibacterial potencies of different related benzoquinones were established against both Gram-negative and Gram-positive bacteria strains. Compounds **6**–**8** with a 2–4 carbon alkyl side chain revealed similar activities against various microbes with MIC ranging from 16 to 256 µg/mL. There was a marked improvement of antibacterial activities with increasing length of the lipophilic chain to 7 as shown by **9** exhibiting low MIC values ranging from 4 to 32 µg/mL.

There was reduced antimicrobial activities with compounds **10** (C9) against most bacteria strains with the lowest activity recorded having MIC ≥ 256 µg/mL against *E. coli* ATCC 8739, AG102, AG100A_Tet_; *S. aureus* A4, A11; *K. pneumoniae* ATCC11296, KP63; *P. stuartii* ATCC29916, NAE16; *E. aerogenes* EA27 and against all strains of MRSA. The activities of compound **11 **(C13) against specific bacteria was comparable to that of **9** especially against the Gram-negative *E. coli* AG100A_Tet_, *K. pneumoniae* KP55, KP63 with MIC values ranging from 16 to 32 µg/mL.

This compound exhibited selective activities against specific Gram-negative bacteria such as *E. coli* AG100A_Tet_, *K. pneumoniae* KP55, KP63 and the Gram-positive MRSA 4, 6, 8 some of which were more potent than chloramphenicol (Table 2).

Increasing chain lengths of the alkyl group from C15 in compound **12** to C19 in **15**–**17** had no effect on further improving the activities of compound **11** (C13) against most bacteria. However, **17** showed selective activities against three strains of MRSA including MRSA4, 6 and 8. Reduction of the two keto groups of **15** and subsequent acetylation at C1, 2, 4, 5 positions as seen in **17** improved the antibacterial activities against specific bacteria including MRSA 4, 6, 8. The MIC values of **17** against MRSA4 and 8 strains were observed to be almost as low as those of the standard drug with MIC values of 32 (vs 8) and 64 (vs 32), respectively (Table 2) while that against MRSA6 was more active than the standard drug (16 (vs 64) µg/mL. This observation could imply that increasing the chain length up to C7 probably results in more profound antibacterial activities. Further increases in chain length had little effect on the improvement of the antibacterial activities but led to selectivity of the drugs towards specific bacteria, as seen in **11**–**13**. The alkyldibenzoquinone, ardisiaquinone B (**18**) with two benzoquinone rings at the end of the alkyl chain (C16) showed improved activities as compared to the monomeric benzoquinone with a similar number of carbon atoms in the lipophilic chain. This compound showed interesting activities against all strains of *E. coli*, *K. pneumoniae*, *P. staurtii* and MRSA. This compound was also potent against *P. aeruginosa* PA124 and *E. aerogenes* EA27 but seemed to be inactive against Gram-positive *S. aureus*.

Compound **19** with two hydroxyl groups, one at C3′and the other in *meta* orientation at C5′and one methoxy group also in *meta* orientation to the hydroxyl group at C3′, without a substituent at C2′ was active against two strains of MRSA 4 and 6. However, interchanging the hydroxyl group at C3′ for a methoxy group and vice versa completely reduced the antimicrobial activities against these two MRSA strains. This may imply that the substitution pattern of the substituents on ring B where at least one hydroxyl group is placed at C3′, an additional hydroxyl groups in *meta* orientation (either at C1′ and 5′), a methoxy group in *meta* orientation to the C3′ hydroxyl group (either at C1′ and 5′) as observed in **19** (hydroxyl at C3′, one *meta* position to have methoxy and the other hydroxyl) is important for minimal activities against the two strains of MRSA. Compound **21** with two methoxy groups and two hydroxyl groups in *meta* orientation was the most active compound of the evaluated chalcones; against the four MRSA strains. The increased antibacterial activities of this compound could be attributed to the additional oxygenation at C2′ position. The basic requirement for activities against MRSA strains (hydroxyl at C3′, one *meta* position to have methoxy and the other hydroxyl) was observed here too. However, more studies need to be undertaken to understand the active scaffold in the chalcone skeleton. Compound **22** with one hydroxyl group at C5′and two methoxy groups at C1′ and C3′ did not exhibit any antibacterial activities against all the strains of bacteria including MRSA most probably due to the lack of the required scaffold. Compound **23** with two hydroxyl group in *meta* position in ring A but was lacking a methoxy group in second *meta* position was inactive against all bacteria tested including the MRSA strains, except for the Gram-negative *E. aerogenes,* ATCC13048, with minimal activity.

Most of the flavones tested were polymethoxylated making them lipophilic and therefore showing minimal or no activities against the bacteria tested. Previous studies have shown that antifungal compounds tend to be more lipophilic compared to antibacterial compounds and hence these compounds should be tested for their antifungal potential (McClure 1975; Omosa et al. 2014). Compound **34** showed minimal activities against specific bacteria strains with MIC values ranging from 128 to 256 µg/mL most probably due to increase in hydrophilicity (Omosa et al. 2014). The flavanones tested were also inactive due to their lipophilicity as shown in previous studies. The clerodanediterpenoids were inactive against all bacteria tested except for hautriwaic acid (Omosa et al. 2014) which showed minimal activity against some bacterial strains. However, its lactone did not exhibit antimicrobial activities ≤256 µg/mL, most probably because of increased lipophilicity. The presence of a free hydroxyl group at C19 in the clerodane skeleton appears to be necessary for minimal activities against *S. aureus* SA 11, 12; *K. pneumoniae* KP55, 63; *P. aeroginosa* PA01 and *E. aerogenes* ATCC 13048 strains. The kaurane type diterpenoids exhibited similar antimicrobial profile as the clerodanes which could be attributed to their lipophilicity.

### Role of efflux pumps in the susceptibility of tested bacteria

In this study the compounds that attained certain threshold of activities including the anthraquinone, **2**; naphthaquinone, **5**; and some benzoquinones, **6**–**10**, **13** and **18** were combined with the efflux pump inhibitor, EPI, PAβN, in order to assess the involvement of efflux in the activities of these compounds. When tested alone compound **2** showed minimal antibacterial activities against some Gram-negative MDR strains including *E. coli* AG100 and 102, as well as *K. pneumoniae* KP 55 and K63 (Table 2). However, there was substantial enhancement of activities of **2** with PAβN. The observed MICs without PAβN against *E. coli* AG102, 100A_Tet_; *K. pneumoniae* KP 55 and K63 were 128, 16, 32, 128 μg/mL. With PAβN the activities improved by >4 fold, exhibiting MICs higher than the standard, chloramphenicol of 2, 4, <2 and 2 μg/mL, respectively (Table 2). This compound which exhibited good antibacterial activities against MDR Gram-positive bacteria alone also showed improved activities in combination with PAβN. However, the improvement was not as substantial as that observed against the Gram-negative bacteria. The naphthaquinone, plumbagin (**5**) which showed good activities even when tested alone exhibited minimal improvement with PAβN as compared to emodin (**2**) except against AG100A_Tet_ with >8 fold increament. These results consistent with those obtained by Kuete et al. (2011). The antimicrobial activities against the Gram-positive MDR strains increased by >4 fold against MRSA 6, 8 and >2 fold for MRSA 4.

The benzoquinones, **6**–**10** and **18** showed enhancement in activity with PAβN most of which were >2 and 4 folds, with **13** having MICs < 10 μg/mL. These data clearly demonstrate that the quinones and chalcones tested are substrates of bacterial efflux pumps and should be combined to EPI in the fight against MDR bacterial infections. The obtained results are therefore consistent to data previously reported by Kuete et al. (2011), who highlighted the role of efflux pumps in the bacterial resistance to natural products.

## Conclusion

In this study, various anthraquinones, naphthoquinones, benzoquinones, flavonoids (chalcones and polymethoxylated flavones) and diterpenoids (clerodanes and kauranes) were explored for their antimicrobial potential against different drug sensitive Gram-negative and Gram-positive bacteria.

The results show that the anthraquinone (**2**), naphthaquinone, plumbagin (**5**) benzoquinones (**11**, **12**, **17**, **18)**, chalcones (**19**, **21)** were active against MRSA bacteria strains with MIC value ranging from 2 to 128 µg/mL. Structure activity relationships of benzoquinones; which has not been carried out in previous studies, showed that antibacterial activities gradually increased with increasing side chain length from 2, 3, 4 for **6**, **7** and **8**, respectively, with optimal activities being realized with C7 (**9**). This study also showed that there is a minimum chain length that is required for maximum activity in the 2,5 dihydroxy-1,4-benzoquinone moieties. This studies showed that the minimum chain length required for optimal antimicrobial activities was C7 (compound **9**) beyond which no marked activity improvement has been observed. Some compounds selectively inhibited the growth of specific but not all bacteria. These studies revealed that the presence of a hydroxyl group at C3′ together with a methoxy group and a second hydroxyl group in meta orientation in ring B of the chalcone skeleton appears to be necessary for minimal activities against MRSA 4 and 6 as elaborated in **19**–**24**.

These data clearly demonstrate that the tested compounds are substrates of bacterial efflux pumps and should be combined to EPI in the fight against MDR bacterial infections. The obtained results are therefore consistent to data previously reported by Kuete et al. (2011), who highlighted the role of efflux pumps in bacterial resistance to natural products.

## Methods

### Reagents and compounds

The chemicals used in antimicrobial assays were chloramphenicol ≥98 % (Sigma-Aldrich, St-Quentin-Fallavier, France) as reference antibiotic and *p*-Iodonitrotetrazolium chloride ≥97 % (INT, Sigma-Aldrich) as microbial growth indicator (Eloff 1998; Mativandlela et al. 2006). Phenylalanine-Arginine-*β*-Naphthylamide (PAβN; Sigma-Aldrich) was used as efflux pumps inhibitor (EPI). Natural products (Figs. 1, 2) used in the study were obtained from the chemical bank of the natural products research laboratory of the Chemistry Department, University of Nairobi, Kenya. Isolation and identification of the compounds in study were previously reported from the following plants; a number of *Rumex* species including; *Rumex dentatus, R. abyssinicus, R. usambarensis, R. bequaertii, R. ruwenzoriensis, R. crispus*; *Plumbago zeylanica,Myrsine africana*, *Maesa lanceolata*, *Rapanea melanphloes*, *Aloe saponaria*, *Erythrina abyssinica*, *Polygonum senegalense*, *Psiadia punctulata*, *Dodonaea angustifolia* and *Senecio roseiflorus*.

### Bacterial strains and culture media

MDR isolates and reference strains of *Escherichia coli*, *Enterobacter aerogenes*, *Pseudomonas aeruginosa*, *Klebsiella pneumoniae* and *Staphylococcus aureus* are summarized in Table 1. Isolates were conserved at 4 °C and were grown on Mueller–Hinton agar for 24 h before minimal inhibitory concentration (MIC) testing. Mueller–Hinton broth (MHB) was used for the susceptibility tests (Kuete et al. 2008).

**Table 1:** Bacterial strains and features

Bacterial strain	Relevant features	Reference
*Escherichia coli*		
ATCC 8739 and ATCC 10536	Reference strains	
AG100	Wild-type *E. coli* K-12	Viveiros et al. (2005)
AG100ATet	ΔacrAB mutant of AG100; TET^R^ owing to acrF gene markedly overexpressed	Elkins and Mullis (2007)
AG102	AG100 over-expressing AcrAB pump	
*Enterobacter aerogenes*		
ATCC 13048	Reference strain	
EA-CM64	CHLR variant obtained from	Ghisalberti et al. (2005)
EA3	ATCC 13048 overexpressing AcrAB pump	Pradel and Pagès (2002)
	Clinical MDR isolate exhibiting energy-dependent	
	Norfloxacin and chloramphenicol efflux with KAN^R^ AMP^R^	
	NAL^R^ STR^R^ TET^R^	
EA27	Clinical MDR isolate exhibiting	
	Energy-dependent NOR and	
	CHL efflux; KAN^R^ AMP^R^ NAL^R^	
	STR^R^ TET^R^	
EA289	KAN-sensitive derivative of EA27	
EA294	EA289 acrA::KAN^R^	
EA289	EA289 tolC::KAN^R^	
*Klebsiella pneumoniae*		
ATCC 11296	Reference strain	
Kp55	Clinical MDR isolate, TET^R^	Chevalier et al. (2000)
Kp63	AMP^R^ ATM^R^ CEF^R^	
	Clinical MDR isolate, TET^R^ CHL^R^	
	AMP^R^ ATM^R^	
*Pseudomonas aeruginosa*		
PA01	Reference strain	
PA124	MDR clinical isolate	Lorenzi et al. (2009)
*Staphyloccocus aureus*		
ATCC1026	Reference strain	
SA3	Clinical Laboratory isolate, sensitive to methicilin	
SA4		
SA11		
SA12	Clinical laboratory isolate, MET^R^	
MRSA 3		Dzoyem et al. (2013)
MRSA 4		
MRSA 6		
MRSA 8		

*KAN* kanamycin, *TET* tetracycline, *CHL* chloramphenicol, *NOR* norfloxacin, *AMP* ampicillin, *MET* methicillin, *NAL* nalidixic acid, *STR* streptomycin, *ATM* aztreonam, *CEF* cefalothin, *R* resistant, *MDR* multidrug-resistant

### Determination of bacterial susceptibility

The MIC determinations on the tested bacteria were conducted using rapid INT colorimetric assay according to described methods (Eloff 1998) with some modifications (Kuete et al. 2007, 2009). First of all, the test samples and reference antibiotic (RA) were dissolved in dimethyl sulphoxide (DMSO)/Mueller–Hinton Broth (MHB) or DMSO/MHB broth. The final concentration of DMSO was lower than 2.5 % and does not affect the microbial growth. The solution obtained was then added to MHB, and serially diluted two fold (in a 96-wells microplate). One hundred microlitrer (100 µL) of inoculum 1.5 × 10^6^ CFU/mL prepared in appropriate broth was then added. The plates were covered with a sterile plate sealer, then agitated to mix the contents of the wells using a plate shaker and incubated at 37 °C for 18 h. Wells containing adequate broth, 100 µL of inoculum and DMSO to at a final concentration of 2.5 % served as negative control. Choramphenicol was used as a RA. The minimum inhibition concentration (MIC) of samples was detected after 18 h incubation at 37 °C, following addition (40 µL) of 0.2 mg/mL of INT and incubation at 37 °C for 30 min. Viable bacteria reduced the yellow dye (TNT) to pink. MIC was defined as the sample concentration that prevented the color change of the medium and exhibited complete inhibition of microbial growth (Eloff 1998). All assays were carried out in triplicate and were repeated three times. To evaluate the role of efflux pumps in the susceptibility of Gram-negative bacteria to the most active compounds, they were tested in the presence of PAβN (at 30 µg/mL) against selected MDR phenotypes (Table 2) and MICs were determined as mentioned above.

**Table 2:** Minimal inhibitory concentrations (MICs) of the studied compounds against the tested bacterial species

Compounds	Bacteria and MIC values in absence and presence of PAβN (in µg/mL) (with 30 mg/mL PAβN^a^)							
	*E. coli*					*K. pneumoniae*		
	ATTC 8739	ATCC 10536	AG 100	AG 102	AG 100A_Tet_	ATCC 11296	KP 55	KP63
Anthraquinones								
**1**	–	–		–	–	–	–	–
**2**	–	–	–	128 (2)	16 (4)	–	32 (<2)	128 (2)
**3**	256	–	–	–	–	–	–	–
**4**	256	–	–	–	–	–	–	–
Naphthoquinones								
**5**	NT	NT	NT	2 (2)	4 (<0.5)	NT	2 (2)	4 (2)
Benzoquinones								
**6**	128	–	258 (128)	128 (64)	258 (128)	64	128 (128)	256 (128)
**7**	256	–	64 (32)	64 (16)	64 (32)	128	64 (64)	64 (32)
**8**	16	64	32 (16)	16 (8)	32 (32)	–	32 (32)	32 (16)
**9**	32	–	4 (4)	4 (<2)	8 (<2)	32	8 (4)	4 (4)
**10**	–	–	32 (8)	32 (16)	64 (64)	256	32 (32)	32 (16)
**11**	–	–	–	–	16	–	32	16
**12**	–	–	–	–	–	–	–	–
**13**	64	–	16 (<2)	8 (<2)	16 (<2)	128	8 (<2)	8 (4)
**14**	–	–	–	–	–	–	–	–
**15**	–	–	–	–	–	–	–	–
**16**	–	–	–	–	–	–	64	–
**17**	–	–	–	–	–	–	–	–
**18**	16	16	256	64 (64)	64 (32)	16	32 (32)	64 (32)
Flavonoids chalcones								
**19**	–	–	–	–	–	–	–	–
**20**	–	–	–	–	–	–	–	–
**21**	–	–	–	–	–	–	–	–
**22**	–	–	–	–	–	–	–	–
**23**	–	–	–	–	–	–	–	–
**24**	–	–	–	–	–	–	–	–
Flavones								
**25**	–	–	–	–	–	–	–	–
**26**	–	–	–	–	–	–	–	–
**27**	–	–	–	–	–	–	–	–
**28**	256	–	–	–	–	–	–	–
**29**	–	–	–	–	–	–	–	–
**30**	–	–	–	–	–	–	–	–
**31**	–	–	–	–	–	–	–	–
**32**	–	–	–	–	–	–	–	–
**33**	–	–	–	–	–	–	–	–
**34**	128	–	–	–	–	128	128	–
Flavanones								
**35**	–	–	–	–	–	–	–	–
**36**	–	–	–	–	–	–	–	–
**37**	–	–	–	–	–	–	–	–
Diterpenoids clerodane type								
**38**	–	–	–	–	–	–	–	–
**39**	–	–	–	–	–	–	–	–
**40**	–	–	–	–	–	–	–	–
**41**	256	–	–	–	–	–	128	256
Kaurane type								
**42**	–	–	–	–	–	–	–	–
**43**	–	–	–	–	–	–	–	–
**44**	–	–	–	–	–	–	–	–
**45**	–	–	–	–	–	–	–	–
**46**	–	–	–	–	–	256	–	–
**47**	256	–	–	–	–	–	256	–
**48**	–	–	–	–	–	–	–	–
CHL	4	16	8	4	32	16	8	32

Compounds	Bacteria and MIC values in absence and presence of PAβN (in µg/mL) (with 30 mg/mL PAβN^a^)								
	*P. aeruginosa*		*P. stuartii*		*E. aerogenes*				
	PA01	PA124	ATCC 29916	−E16	ATCC 13048	EA-CM64	EA3	EA27	EA289
Anthraquinones									
**1**	–	–	–	–	–	–	–	–	–
**2**	–	–	–	–	–	–	–	–	128
**3**	–	–	–	–	–	–	–	–	–
**4**	–	–	–	–	–	–	–	–	–
Naphthoquinones									
**5**	–	–	–	–	–	NT	NT	64	64
Benzoquinones									
**6**	32	128 (128)	64	128	128 (32)	128 (128)	–	128 (32)	
**7**	32	64 (32)	128	32	64 (16)	64 (32)	–	64 (32)	
**8**	16	64 (16)	32	64	32 (8)	32 (32)	128	32 (16)	
**9**	16	16 (4)	32	256	4 (4)	64 (4)	–	8 (<2)	
**10**	16	64 (32)	–	256	8	64 (32)	–	64 (8)	
**11**	–	–	–	–	–	–	–	–	
**12**	–	–	–	–	–	–	–	–	
**13**	32	16 (4)	128	128	8 (<2)	16 (8)	–	8 (<2)	
**14**	–	–	–	–	–	–	–	–	
**15**	–	–	–	–	256	–	–	–	
**16**	–	–	–	–	–	–	–	–	
**17**	–	–	–	–	–	–	–	–	
**18**	–	256	16	16	–	–	32	16-	
Flavonoids chalcones									
**19**	–	–	–	–	–	–	–	–	–
**20**	–	–	–	–	256	–	–	–	–
**21**	–	–	–	–	–	–	–	–	128
**22**	–	–	–	–	–	–	–	–	–
**23**	–	–	–	–	256	–	–	–	–
**24**	–	–	–	–	–	–	–	–	–
Flavones									
**25**	–	–	–	–	–	–	–	–	–
**26**	–	–	–	–	–	–	–	–	–
**27**	–	–	–	–	–	–	–	–	–
**28**	–	–	–	–	–	–	–	–	–
**29**	–	–	–	–	–	–	–	–	–
**30**	–	–	–	–	–	–	–	–	–
**31**	–	–	–	–	–	–	–	–	–
**32**	–	–	–	–	–	–	–	–	–
**33**	–	–	–	–	–	–	–	–	–
**34**	128	–	–	–	128	–	–	–	–
Flavanones									
**35**	–	–	–	–	–	–	–	–	–
**36**	–	–	–	–	–	–	–	–	–
**37**	–	–	–	–	–	–	–	–	–
Diterpenoids clerodane type									
**38**	–	–	–	–	–	–	–	–	–
**39**	–	–	–	–	–	–	–	–	–
**40**	–	–	–	–	256	–	–	–	–
**41**	128	–	–	–	128	–	–	–	–
Kaurane type									
**42**	–	–	–	–	–	–	–	–	–
**43**	–	–	–	–	–	–	–	–	–
**44**	–	–	–	–	256	–	–	–	–
**45**	–	–	–	–	–	–	–	–	–
**46**	–	–	–	–	–	–	–	–	–
**47**	–	–	–	–	256	–	–	–	–
**48**	–	–	–	–	64	–	–	–	–
CHL	8	128	16	32	4	256	32	64	128

Compounds	Bacteria and MIC values in absence and presence of PAβN (in µg/mL) (with 30 mg/mL PAβN^a^)									
	*S. aureus*									
	ATCC 1026	SA3	SA4	SA11	SA12	ATCC 1026	MRSA3	MRSA4	MRSA6	MRSA8
Anthraquinones										
**1**	–	–	–	–	–	–	–	–	–	–
**2**	–	–	–	–	–	–	–	4 (2)	4 (0.5)	4 (1)
**3**	–	–	–	–	–	–	–	–	–	–
**4**	–	–	–	–	–	–	–	–	–	–
Naphthoquinones										
**5**	NT	NT	NT	NT	NT	NT	64	2 (1)	2 (<0.5)	2 (<0.5)
Benzoquinones										
**6**	256	64	256	64	128	256	–	–	–	–
**7**	–	64	256	64	128	–	–	–	–	–
**8**	–	32	256	16	32	–	–	–	–	–
**9**	–	4	128	16	16	–	–	–	–	–
**10**	–	4	256	64	16	–	–	–	–	–
**11**	–	–	–	–	–	–	–	4	32	64
**12**	–	–	–	–	–	–	–	128	–	64
**13**	–	4	–	128	16	–	–	–	–	–
**14**	–	–	–	–	–	–	–	–	–	–
**15**	–	–	–	–	–	–	–	–	–	–
**16**	–	–	–	–	–	–	–	–	–	–
**17**	–	–	–	–	–	–	–	32	16	64
**18**	–	–	–	–	–	–	–	64 (64)	64 (64)	32 (32)
Flavonoids Chalcones										
**19**	–	–	–	–	–	–	–	64	128	–
**20**	–	–	–	–	–	–	–	–	–	–
**21**	–	–	–	–	–	–	128	16	32	64
**22**	–	–	–	–	–	–	–	–	–	–
**23**	–	–	–	–	–	–	–	–	–	–
**24**	–	–	–	–	–	–	–	128	–	–
Flavones										
**25**	–	–	–	–	–	–	–	–	–	–
**26**	–	–	–	–	–	–	–	–	–	–
**27**	–	–	–	–	–	–	–	–	–	32
**28**	–	–	–	–	–	–	–	–	–	–
**29**	–	–	–	–	–	–	–	–	–	–
**30**	–	–	–	–	–	–	–	–	–	–
**31**	–	–	–	–	–	–	–	–	–	–
**32**	–	–	–	–	–	–	–	–	–	–
**33**	–	–	–	–	–	–	–	32	–	–
**34**	–	–	–	128	256	–	–	–	–	–
Flavanones										
**35**	–	–	–	–	–	–	–	–	–	–
**36**	–	–	–	–	–	–	–	–	–	–
**37**	–	–	–	–	–	–	–	–	–	–
Diterpenoids clerodane type										
**38**	–	–	–	–	–	–	–	–	–	–
**39**	–	–	–	–	–	–	–	128	–	–
**40**	–	–	–	–	–	–	–	–	–	–
**41**	–	–	–	64	128	–	–	–	–	–
Kaurane type										
**42**	–	–	–	–	–	–	–	–	–	–
**43**	–	–	–	–	–	–	–	–	–	–
**44**	–	–	–	–	–	–	–	–	–	–
**45**	–	–	–	–	–	–	–	–	–	–
**46**	–	–	–	–	–	–	–	–	–	–
**47**	–	–	–	–	–	–	–	–	–	–
**48**	–	–	–	–	–	–	–	–	–	–
CHL	128	4	8	4	8	128	–	8	64	32

*NT* not tested because the sample was insufficient, – sample not active up to 256 mg/L, *CHL* chloramphenicol

^a^The MIC of PAβN was 64 μg/mL for AG100A and >256 mg/L for other *E. coli*, *E. aerogenes*, *K. pneumoniae* and *P. aerogenes* strains

## References

[CR1] Alekshun MN, Levy SB (2007). Molecular mechanisms of antibacterial multidrug resistance. Cell.

[CR2] Brice HE (1955). Antibacterial substances produced by flowering plants. Aust J Exp Biol Med.

[CR3] Chang CH, Lin CC, Yang JJ, Namba T, Hattori M (1996). Anti-inflammatory effects of emodin from ventilago leiocarpa. Am J Chin Med.

[CR4] Chevalier J, Pagès J-M, Eyraud A, Malléa M (2000). Membrane permeability modifications are involved in antibiotic resistance in *Klebsiella pneumoniae*. Biochem Biophys Res Commun.

[CR5] Dagne E, Casser I, Steglich W (1992). Aloechrysone, a dihydroanthracenone from *Aloe berhana*. Phytochem.

[CR6] Dagne E, Van Wyk B-E, Mueller M, Steglich W (1996). Three dihydroanthracenones from *Gasteria bicolor*. Phytochem.

[CR7] Davin-Regli A, Bolla JM, James CE, Lavigne JP, Chevalier J, Garnotel E, Molitor A (2008). Membrane permeability and regulation of drug “influx and efflux” in enterobacterial pathogens. Curr Drug Targets.

[CR8] De Clercq E (2001). New developments in anti-HIV chemotherapy. Pharmacol.

[CR9] Durga R, Sridhar P, Polasa H (1990). Effect of plumbagin on antibiotic resistance in bacteria. Indian J Med Res.

[CR10] Dzoyem JP, Hamamoto H, Ngameni B, Ngadjui BT, Sekimizu K (2013). Antimicrobial action mechanism of flavonoids from *Dorstenia* species. Drug Discov Ther.

[CR11] Elkins CA, Mullis LB (2007). Substrate competition studies using whole-cell accumulation assays with the major tripartite multidrug efflux pumps of *Escherichia coli*. Antimicrob Agents Chemother.

[CR12] Eloff JN (1998). A sensitive and quick microplate method to determine the minimal inhibitory concentration of plant extracts for bacteria. Planta Med.

[CR13] Fairbairn JJW, El-Muhtadi FJ (1972). Chemotaxonomy of anthraquinones in *Rumex*. Phytochem.

[CR14] Ghisalberti D, Masi M, Pagès J-M, Chevalier J (2005). Chloramphenicol and expression of multidrug efflux pump in *Enterobacter aerogenes*. Biochem Biophys Res Commun.

[CR15] Gujar GT (1990). Plumbagin, a naturally occurring naphthoquinone. Its pharmacological and pesticidal activity. Fitoterapia.

[CR16] Hatano T, Uebayashi H, Ito H, Shiota S, Tsuchiya T, Yoshida T (1999). Phenolic constituents of Cassia seeds and antibacterial effect of some naphthalenes and anthraquinones on methicillin-resistant *Staphylococcus aureus*. Chem Pharm Bull.

[CR17] Jeu L, Piacenti FJ, Lyakhovetskiy AG, Fung HB (2003). Voriconazole. Clin Ther.

[CR18] Jeyachandran R, Mahesh A, Cindrella L, Sudhakar S, Pazhanichamy K (2009). Antibacterial activity of Plumbagin and root extracts of *Plumbago zeylanica* L.. Acta Biol Cracov Bot.

[CR19] Juma BF, Yenesew A, Midiwo JO, Waterman PG (2001). Flavones and phenylpropenoids in the surface exudate of *Psiadia punctulata*. Phytochem.

[CR20] Juma BF, Midiwo JO, Yenesew A, Waterman PG, Heydenreich M, Peter MG (2006). Three ent-trachylobane diterpenes from the leaf exudates of *Psiadia punctulata*. Phytochem.

[CR21] Kerubo LO, Midiwo JO, Derese S, Langat MK, Akala HA, Waters NC, Peter M, Heydenreich M (2013). Antiplasmodial activity of compounds from the surface exudates of *Senecio roseiflorus*. Nat Prod Commun.

[CR22] Khurana SK, Krishnamoorthy V, Seshadri TR (1972). Mass spectral analysis of the pigments from *Embelia ribes* and *Connarus monocarpus* Linn. Cur Sci.

[CR23] Kimberlin DW, Whitley RJ (1996). Anti-viral resistance: mechanisms, clinical significance, and future implications. J Antimicrob Chemother.

[CR24] Kuete V (2010). Potential of Cameroonian plants and derived products against microbial infections: a review. Planta Med.

[CR25] Kuete V, Efferth T (2010). Cameroonian medicinal plants: pharmacology and derived natural products. Front Pharmacol.

[CR26] Kuete V, Wabo GF, Ngameni B, Mbaveng AT, Metuno R, Etoa F-X, Ngadjui BT, Beng VP, Meyer JJM, Lall N (2007). Antimicrobial activity of the methanolic extract, fractions and compounds from the stem bark of *Irvingia gabonensis* (Ixonanthaceae). J Ethnopharmacol.

[CR27] Kuete V, Wansi JD, Mbaveng AT, Sop MK, Tadjong AT, Beng VP, Etoa F-X, Wandji J, Meyer JJM, Lall N (2008). Antimicrobial activity of the methanolic extract and compounds from *Teclea afzelii* (Rutaceae). S Afr J Bot.

[CR28] Kuete V, Nana F, Ngameni B, Mbaveng AT, Keumedjio F, Ngadjui BT (2009). Antimicrobial activity of the crude extract, fractions and compounds from stem bark of *Ficus ovata* (Moraceae). J Ethnopharmacol.

[CR29] Kuete V, Alibert-Franco S, Eyong KO, Ngameni B, Folefoc GN, Nguemeving JR, Tangmouo JG, Fotso GW, Komguem J, Ouahouo BMW, Bolla J-M, Chevalier J, Ngadjuic BT, Nkengfack AE, Pagès J-M (2011). Antibacterial activity of some natural products against bacteria expressing a multidrug-resistant phenotype. Int J Antimicrob Ag.

[CR30] Lorenzi V, Muselli A, Bernardini AF, Berti L, Pagès JM, Amaral L (2009). Geraniol restores antibiotic activities against multidrug-resistant isolates from Gram negative species. Antimicrob Agents Chemother.

[CR31] Manguro LOA, Midiwo JO, Kraus W, Ugi I (2003). Benzoquinone derivatives of *Myrsine africana* and *Maesa lanceolata*. Phytochem.

[CR32] Mativandlela SPN, Lall N, Meyer JJM (2006). Antibacterial, antifungal and antitubercular activity of (the roots of) *Pelargonium reniforme* (CURT) and *Pelargonium sidoides* (DC) (Geraniaceae) root extracts. S Afr J Bot.

[CR33] McClure JW (1975). Physiology and functions of flavonoids. The flavonoids.

[CR34] Mehandale AR, Rao AR, Shaikh IN, Venkataraman K (1968). Desoxyerythrolaccin and laccaic acid D. Tetrahedron Lett.

[CR35] Melo AM, Jardim M, Santana C, Lacet Y, Filho J, Lima I, Leoncio OG (1974). First observations on the topical use of primin, plumbagin and mayteni in patients with skin cancer. Rev I Antibiot.

[CR36] Merian R, Schlittler E (1948). Über das vorkommen von Embelin in der Familie der Myrsinaceen. Helv Chim Acta.

[CR37] Midiwo JO, Rukunga GM (1985). Distribution of Anthraquinone Pigments in *Rumex* species of Kenya. Phytochem.

[CR38] Midiwo JO, Matasi JJ, Wanjau OM, Mwangi RW, Waterman PG, Wollenweber E (1990). Anti-feedant effects of surface accumulated flavonoids of *Polygonum senegalense*. B Chem Soc Ethiopia.

[CR39] Midiwo JO, Gikonyo NK, Wanjau DO, Matasi JJ, Waterman PG (1992). Flavonoids of *Polygonum senegalense* (Meisn) Part II: more surface and internal tissue flavonoid aglycones. B Chem Soc Ethiopia.

[CR40] Midiwo JO, Owuor FAO, Juma BF, Waterman PG (1997). Diterpenes from the leaf exudate of *Psiadia punctulata*. Phytochem.

[CR41] Munavu RM, Mudamba LO, Ogur JA (1984). Isolation and characterization of the major anthraquinone pigments from *Rumex abysinica*. Planta Med.

[CR42] Murthy VK, Rao TVP, Venkateswarlu V (1965). Chemical examination of *Ardisia macrocarpa* Wall. Tetrahedron.

[CR43] Nakahara K, Roy MK, Ono H, Maeda I, Ohnishi-Kameyama M, Yoshida M, Trakoontivakorn G (2003). Prenylated flavanones isolated from flowers of *Azadirachta indica* (the neem tree) as antimutagenic constituents against heterocyclic amines. J Agri Food Chem.

[CR44] Nikaido H (2009). Multidrug resistance in bacteria. Annu Rev Biochem.

[CR45] Ogawa H, Natori S (1965). Hydroxybenzoquinones from Myrsinaceae Plants. I. Reconfirmation of the Structure of Maesaquinoes and Isolation of Acetylmaesaquinone from *Maesa japonica* MORITZI. Chem Pharm Bull.

[CR46] Ogawa H, Natori S (1968). Hydroxybenzoquinones from myrsinaceae plants-II: distribution among myrsinaceae plants in Japan. Phytochem.

[CR47] Ogawa H, Natori S (1968). Hydroxybenzoquinones from Myrsinaceae plants. III. The structures of 2-hydroxy-5-methoxy-3-pentadecenylbenzoquinone and Ardisiaquinones A, B and C from *Ardisia* spp.. Chem Pharm Bull.

[CR48] Omosa LK, Midiwo JO, Derese S, Yenesew A, Peter MG, Heydenreich M (2010). neo-Clerodane diterpenoids from the leaf exudate of *Dodonaea angustifolia*. Phytochem Lett.

[CR49] Omosa LK, Amugune B, Ndunda B, Milugo TK, Heydenreich M, Yenesew A, Midiwo JO (2014). Antimicrobial flavonoids and diterpenoids from *Dodonaea angustifolia*. S Afr J Bot.

[CR50] Poole K (2001). Overcoming anti-microbial resistance by targeting resistance mechanisms. J Pharm Pharmacol.

[CR51] Pradel E, Pagès J-M (2002). The AcrAB–TolC efflux pump contributes to multidrug resistance in the nosocomial pathogen Enterobacter aerogenes. Antimicrob Agents Chemother.

[CR52] Sidhu GS, Sankaram A, Ali SM (1968). Extractives from *Diospyros* Species. 3. New Naphthaquinones + Naphthols from Heartwood of Diospyros melanoxylon Roxb. Indian J Chem.

[CR53] Silver L, Bostian K (1990). Screening of natural products for anti-microbial agents. Eur J Cli Microbiol Infect Dis.

[CR54] Taylor PW, Stapleton PD, Luzio JP (2002). New ways to treat bacterial infections. Drug Discov Today.

[CR55] Viveiros M, Jesus A, Brito M, Leandro C, Martins M, Ordway D (2005). Inducement and reversal of tetracycline resistance in *Escherichia coli* K-12 and expression of proton gradient-dependent multidrug efflux pump genes. Antimicrob Agents Chemother.

[CR56] Wouters J, Verhecken A (1987). The chemical nature of flavokermesic acid. Tetrahedron Lett.

[CR57] Wu L, Yang C, Yang L (2009). Synthesis of 2-hydroxy-5-methoxy-3-alkyl-1, 4-benzoquinones. Asian J Chem.

[CR58] Yagi A, Yamanouchi M, Nishioka I (1978). Biosynthetic relationship between tetrahydroanthracene and anthraquinone in *Aloe saponaria*. Phytochem.

[CR59] Yoshihir K, Sakaki S, Ogawa H, Natori S (1968). Hydroxybenzoquinones from Myrsinaceae plants. 4. Further confirmation of structures of ardisiaquinones and some observations on alkylaminobenzoquinone derivatives. Chem Pharm Bull.

[CR60] Yuan LIU, Chao LIU (2007). Determination the content of plumbagin in *Plumbago zeylanica* L. from different areas by RP-HPLC [J]. Chin Pharm J.

[CR61] Zhang H, Guo Z, Wu N, Xu W, Han L, Li N, Han Y (2012). Two novel naphthalene glucosides and an anthraquinone isolated from *Rumex dentatus* and their antiproliferation activities in four cell lines. Molecules.

